# Remote online open book examinations: through the lenses of faculty and students in health professions programmes

**DOI:** 10.1186/s12909-023-04368-0

**Published:** 2023-06-02

**Authors:** Hui Meng Er, Pei Se Wong, Vishna Devi Nadarajah

**Affiliations:** 1 IMU Centre for Education and School of Pharmacy, International Medical University, Kuala Lumpur, Malaysia; 2 IMU Centre for Education and School of Medicine, International Medical University, Kuala Lumpur, Malaysia

**Keywords:** Remote, Online, Open book, Examination, Health professions

## Abstract

**Background:**

Open book examinations have been used to assess students’ higher order cognitive skills. These examinations can be conducted online remotely with the advancement of technology. However, there are concerns regarding its validity and reliability particularly if the examinations are not proctored. The objective of this study was to explore the perceptions of faculty and students in health professions programmes about remote online open book examinations (ROOBE).

**Methods:**

Semi-structured interviews were conducted among 22 faculty staff who were involved in ROOBE in health professions programmes. All interviews were audio recorded, transcribed verbatim and analysed using a thematic analysis approach. The perceptions of 249 medical students were obtained using an online questionnaire after they completed ROOBE.

**Results:**

The faculty agreed that open book examinations could promote students’ higher order cognitive skills and reduce students’ stress. However, they were concerned about students’ academic integrity during non-invigilated ROOBE which could affect recognition by accreditation and professional bodies. The shift from traditional practice of closed-book examinations to ROOBE required change management with the support of guidelines and faculty training.

Majority of the students claimed that the examinations were challenging as they assessed their ability to apply knowledge in real world problems. Nevertheless, they preferred ROOBE due to less anxiety and memorisation, and more emphasis on problem solving skills. The shortcomings were insufficient time for information searching during examinations and uncertainty in preparedness for future practice as they focused less on memorisation of factual knowledge during examination preparation. Cheating among peers and internet instability during non-invigilated ROOBE were the concerns highlighted by some students.

**Conclusions:**

Faculty and students expressed favourable views about ROOBE in promoting higher order cognitive skills. Adequate technological support was essential during ROOBE. While there was a need to address issues related to academic integrity, ROOBE could be included as an authentic assessment within the systems of assessment.

## Background

Open book examination (OBE) has gained popularity in the recent years as an appropriate assessment tool to address the rapid expansion and accessibility of knowledge especially in the biomedical field. OBE has been recommended as an alternative or complementary assessment tool to Closed Book Examinations (CBE) in view of its advantages and values. For example, OBE emphasises learning outcomes related to higher order cognitive skills such as deep learning and critical thinking, and use of relevant and verified references [1–3]. These skills are much needed to future professional roles and practice [4, 5]. A study by Ramamurthy et al. [6] on the pharmacy students’ perception of OBE and its impact on performance and learning approach suggested that OBE was preferred by pharmacy students as it placed emphasis on problem solving and analytical thinking. They experienced less anxiety in OBE as less memorisation was required, which could have contributed to their better performance in OBE compared to closed book examination (CBE). However, there was no significant difference in the pharmacy students’ learning approach between OBE and CBE. Meanwhile, it was reported that CBE stimulated deep learning approach among Years 2 and 3 medical students more than OBE [7]. According to the systematic review conducted by Durning et al. that compared the utility of OBE and CBE, there was insufficient evidence for the exclusive use of either tool [8]. More recent studies provide some additional insight, for example, Davies et al. [9] showed that second year medical students obtained higher scores in OBE (where students had access to resources) compared to CBE, with greater difference on factual recall compared to application questions. In another study by Al-Sharbatti et al. [10], there was no significant difference in the medical and dental students’ performance in higher cognitive level items in OBE and CBE despite access to electronic resources during OBE. The varied findings in literatures strongly suggest that the student performance in OBE or CBE is dependent on the test item characteristics and cognitive level of the test constructs. This also highlights the importance of quality assurance of assessments regardless of the tool.

Conventionally, examinations are conducted onsite (normally in campus) under in-person invigilation. This can be in the form of paper-based or online examinations. The advancement of technology and internet accessibility has enabled online examinations to be conducted remotely whereby the students can undertake the online examination from any location outside the campus. The similarities and differences between the two modes of examinations are shown in Table 1.

**Table 1:** Comparison of conventional onsite and remote online examinations

	**Conventional onsite online examinations**	**Remote online examinations**
**Examination site**	Students undertake examinations in campus	Students undertake examinations outside campus (eg. from home)
**Presence of invigilation during examination**	Invigilated in-person	Invigilated (proctored using computer software) or non-invigilated
**Format of examination**	CBE or OBE	CBE or OBE
**Mode and duration of examination**	All students take the examination online at the specified time and within a fixed duration	Students take the examination online at the specified time or within a window period and submit answers within a fixed duration

The Covid-19 pandemic has inadvertently catalysed the acceptance of online learning and assessments, including the use of online OBE in medical education [11, 12]. Remote online open book examinations (ROOBE) were widely used during the COVID-19 pandemic due to campus lockdown. While ROOBE is aligned with the concept of flexible learning, its validity and reliability are often challenged due to concerns such as student cheating through collaborating with each other during a non-invigilated examination [13], or students using latest AI related technologies to answer questions [14, 15]. Consequently, online artificial intelligence (AI)-based proctoring tools are sprouting in the markets in the past two years to deter academic misconduct during online examinations [16]. Nevertheless, none of these are completely foolproof. Besides, the associated technical, ethical and other concerns remain [17].

The challenges affecting ROOBE makes selecting an assessment method a complex process. The assessment utility formula proposed by Van der Vleuten [18] continues to be relevant whereby vigorous assessment systems shape the validity, reliability, educational impact, practicability, cost, and acceptability of assessment. The extent to which all stakeholders find the assessment to be appropriate and reasonable is an important consideration when selecting the assessment tool. Although the opinions and attitudes of students and faculty are often not the main consideration in designing assessments, their acceptability influences the implementation and sustainability of assessment procedures. Therefore, this study was designed to explore the perceptions of faculty and students of health professions programmes on ROOBE without invigilation, with the assessment utility formula mentioned above as the conceptual framework. The findings of this study can help identify both enablers and key improvement areas in the design and implementation of ROOBE.

## Methods

### Ethics approval

The study was approved by the International Medical University Joint Committee on Research and Ethics [IMU 483/2020]. The study objectives and information were explained to the participants and their consent was obtained before data collection.

### Study design

The study was conducted at the International Medical University (IMU), Malaysia. It is a private university that offers a range of undergraduate and postgraduate health professions programmes.

The study used a mixed-method design consisting of qualitative study involving faculty interviews on their perceptions of ROOBE, and quantitative data collection and analysis on students’ perceptions of ROOBE. Qualitative approach was chosen to gather faculty perceptions as the design enabled an in-depth insight into opinions at various stages of design and implementation of ROOBE. A quantitative approach was chosen for the student data collection in order to reach out to a larger student population.

### Study participants

Participants for semi-structured interviews were teaching faculty purposively recruited from various schools in the university (i.e. School of Medicine, School of Dentistry, School of Pharmacy, School of Health Sciences and School of Postgraduate Studies) with at least 5 years of teaching experience and held administrative roles such as module coordinators or assessment coordinators. They were recruited for interviews through an e-mail invitation explaining the objectives of the study and data confidentiality. Consenting participants were invited to attend the interviews.

Semi-structured interviews were conducted from July to November 2020 with 22 faculty members (8 males, 14 females) from the School of Medicine (*n* = 4), School of Pharmacy (*n* = 7), School of Dentistry (*n* = 2), School of Health Sciences (*n* = 6), and School of Postgraduate Studies (*n* = 3). Among them, 6 had 5–10 years of teaching experience; 12 with 11–19 years of teaching experience; 4 with 20–25 years of teaching experience.

Medical students (Years 1 to 3) who had prior experience with CBE and ROOBE in the Bachelor of Medicine and Bachelor of Surgery (MBBS) programme were invited to complete the student survey online using Survey Monkey®. The students undertook ROOBE in the format of multiple-choice questions (the duration given to attempt each question was 1-1.5 min) between April 2020 and February 2021. The modules involved were integrated body system modules including Cardiovascular, Respiratory and Haematology systems in Semester 2 (Year 1); Gastrointestinal, Endocrine and Renal systems in Semester 3 (Year 2); Reproductive, Musculoskeletal and Nervous systems in Semester 4 (Year 2). There is at least one ROOBE in each module. The students had undertaken at least three ROOBE as in-course or final assessments when the survey on students’ perceptions of ROOBE was conducted in March 2021. Prior to the COVID-19 pandemic, theory examinations, mostly CBE, were conducted in the campus under in-person invigilation using the university’s Online Assessment System (OAS). Since the outbreak of COVID-19 in April 2020, ROOBE has been introduced for in-course and final assessments. As with the pre-COVID-19 arrangement, students undertook these examinations online at the specified time and submitted answers within a fixed duration. Since November 2020, online invigilation was introduced to ROOBE, whereby the students were invigilated through Zoom application with their microphones and video cameras turned on using a second device, while taking ROOBE on the OAS. To familiarise the students with the conduct and types of questions in ROOBE, mock examinations were arranged at least once before the students’ first encounter with summative ROOBE in their programmes.

### Data collection and analysis

Semi-structured interviews with the faculty participants were conducted online via Microsoft Teams by one of the researchers in the study (PSW). Each interview took about 30–60 min. The interviews were guided by a set of pre-determined open questions as shown in Table 2, which were designed based on the assessment utility formula [18]. The interview guide was pilot tested with a faculty. Issues related to the questions were discussed and minor amendments were made to the interview guide following the pilot testing. All interviews were audio recorded with the consent of the participants and transcribed verbatim. Thematic analysis was used to analyse the interview data [19]. The researchers (HME and PSW) independently coded the interviews, following which the codes were discussed. Coded data were analysed, compared, and combined to form themes. The themes were further discussed among all the researchers and refined to ensure reliability. Data saturation was obtained after 20 interviews. Two more interviews were conducted to confirm that no new theme was generated.

**Table 2:** Semi-structured interview guide

Construct	Discussion Prompts
Perceptions of remote online open book examination	• What is/are your role(s) in the remote online open book examination? • What are your perceptions of the remote online open book examination?
Perceptions of the design of remote online open book examination	• What were the reasons that led you/your teaching team to decide to offer the exam in an online open book format? If the reason given is due to situation: Would you have made this decision if not because of the situation? • Tell me about your experiences (positive and negative) in designing the remote online open book examination • What do you considered when you design and plan the remote online open book examination? • Did you face any challenge when designing the remote online open book exam? Elaborate if so • What support did you receive when designing the remote online open book examination? • What has helped in the designing the remote online open book examination? • How could the design or planning of remote online open book examination be improved?
Perceptions of the conduct of remote online open book examination	• Was the exam invigilated/proctored? If yes, how was it done? • How well do you think the remote online open book examination been conducted? • Did they students encounter any issues during the exam? What types of issues? • Do you/your colleagues/students who were involved in the design and conduct of the exams have any concerns on the conduct? What are their concerns? • How could the conduct of remote online open book examination be improved?
Perceptions of the future of remote online examination	• Would you consider online open book exam in future? Why?. Explore all possibilities – Remote (yes/no) and on campus (yes/no) • What do you think are the impacts of online open book exam on students’ learning? • What is your view on the future of remote online open book examination?

The students’ perceptions of ROOBE were collected using a questionnaire that comprised of 18 open and closed-ended questions (Table 3). The questionnaire was pilot tested by a few students who were not involved in the study to confirm its face validity. The student responses in the questionnaire were analysed.

**Table 3:** Questionnaire for students’ perceptions of ROOBE

1. What device(s) did you use to take the online remote open book examination? You can select more than one options. □ Desktop □ Laptop □ Smart tablet □ Smart phone □ Other device(s). Please state: ________________
2. Was the examination invigilated (i.e. were you supervised during the conduct of the examination)? □ Yes □ No If yes, please state the method of invigilation (eg. closed circuit television, computer software with webcam etc.): ________________________ If no, please state if you have any concern that the online remote open book examination was not invigilated _________________________________________________________________
3. Were the questions in the online remote open book examination challenging? □ Not challenging at all □ Quite challenging □ Very challenging
4. The questions in the online open book examination test the intended learning outcomes of the modules/courses. □ Strongly disagree □ Disagree □ Agree □ Strongly agree
5. The questions in the online open book examination test what I learn during the teaching and learning activities in the modules/courses. □ Strongly disagree □ Disagree □ Agree □ Strongly agree
6. The questions in the online open book examination require me to apply my knowledge in real life situations. □ Strongly disagree □ Disagree □ Agree □ Strongly agree
7. The formative assessment in the modules/courses prepare me for the online open book examination. □ Strongly disagree □ Disagree □ Agree □ Strongly agree
8. The mock examination was helpful in preparing me for the actual examination. □ Strongly disagree □ Disagree □ Agree □ Strongly agree
9. Did you refer to any learning materials/resources during the online remote open book examination? □ Yes □ No If Yes, please answer Questions 10–13 If No, please go to Question 14
10. What types of learning materials/resources did you refer to during the online remote open book examination? You can select more than one options. □ Lecture notes (or Powerpoint slides) provided by the lecturers □ Notes prepared by yourself □ Textbooks □ Other learning resources provided by the lecturers (eg. reading article) □ Internet resources □ Others. Please state: _____________________________________

## Results

### Faculty perceptions of ROOBE

A deductive approach was adopted in the thematic analysis of the faculty interviews. The themes, defined by the utility formula of assessment [18] i.e. validity, reliability, educational impact, acceptability and cost effectiveness, and sub-themes were identified from the interview codes as presented in Table 4. Examples of the interviewees’ quotes for each theme and sub-theme are available in Table 5.

**Table 4:** Themes and sub-themes based on faculty interviews

Themes	Sub-themes	Total number of quotes
Validity	Assessment blueprinting	76
	Faculty competencies	15
	Quality assurance	33
Reliability	Student performance	8
	Standard setting	5
Educational impact	Promotion of higher order cognitive skills	28
Acceptability	Recognition by stakeholders	7
	Change management (faculty and student readiness)	164
	Impact of COVID-19 pandemic	26
	Academic integrity	57
Cost	IT softwares	26
	Internet connection	20
	Technical support	8
	Faculty time	11

**Table 5:** Examples of interviewees’ quotes under the sub-themes

Themes	Sub-themes	Examples of quotes
Validity	Assessment blueprinting	*“I think open book is the way forward for us because we are teaching professional courses which involves a lot of decision making … decision making ability is one of the key outcomes. So that should really be tested right from the beginning.” (P8)* *“For certain modules, practice related modules, I think this is a suitable exam format. But of course not all modules can do that. I would say majority of the modules, especially those lower semesters which are mainly knowledge based, I think conventional face-to-face is still more suitable.” (P3)*
	Faculty competencies	*“Setting the question is a major challenge for the faculty. If every faculty can be trained to set higher order thinking questions, then I think the faculty are ready.” (P10)* *“During the vetting session, we sat down together and looked at the appropriateness, the level and the rubric. So we always checked that and we improved (the questions) during the vetting session.” (P21)*
	Quality assurance	*“I think having the vetting is really essential, because we were able to help each other a lot during the vetting and we were really able to, at least at the department level we're definitely able to help each other make better questions” (P2)*
Reliability	Student performance	*“As a teacher, of course initially I doubt the reliability. But after conducting the exercise, I think it is quite okay. I mean the result is quite reliable” (P5)*
	Standard setting	*“When we did the standard setting, we specifically remind the faculty to keep in mind that these are open book exams, meaning the students can access the resources to answer those questions.” (P1)*
Educational impact	Promotion of higher order cognitive skills	*“If we just test on the recall questions then we expect the students to just do a search, find and then if they find the answers they will just click on the correct answer. But if we are testing on the higher order questions, then the students they will realise that "now I need to study and I need to understand the concept". So a total different game that they will have to play now.” (P10)* *“In the future I think we are going that direction. So our values to society then comes into the equation … the strategizing, analysis, individualisation to different situations, and that is what we call higher order … And if you want to survive as a university we must be competitive and our students must all be prepared for this future.” (P7)*
Acceptability	Recognition by stakeholders	*“I'm just wondering from an external point of view, let's say if you have a student who graduated by taking this type of open book exam, I don't know will there be any biasness from the employers, can they rely on the scroll received by that student?” (P12)*
	Change management (faculty and student readiness)	*“Move forward, I think if we think positively, I think the change… I think we can actually embrace the change. I think it's a good way, I think if it doesn't happen we also would not know how things will turn out. So as I said it's a learning phase for me as well as the students, so I think it's a good way.” (P13)* *“There are enough literatures to support that these open book exams do work, and they can actually work better for questions where critical analysis is required.” (P4)* *“I think the contingency plan, so a more complete contingency plan… so that like when this happens, what is (plan) B and C for different scenarios that we actually encounter, so it will be nice if this happens, immediately we know what to do.” (P6)* *“Initially I think the students were very resistant about open book exams. They were concerned, I think their idea is that when you are having an open book exam, meaning that your questions will be tougher and they don't know how to answer, meaning that they cannot find the answers in the textbook at all.” (P10)* *“We try to prepare them before they sit for the paper. You know, on what's expected, so during the tutorial, we do give some cases so that they can practise on to help them. Some are able to catch up, so when they respond to the OBA questions, they are okay. But some, not able to….. I think it's no harm to proceed with open book as long as we prepare them well ahead, you know. And I think they should be okay….. I think we can but we need to do it slowly. Not too drastic, you know.” (P13)*
	Impact of COVID-19 pandemic	*“We never really took up that opportunity to convert them to all open book assessment…I think this COVID-19 pandemic has open up those doors.” (P1)*
	Academic integrity	*“To be honest, every time when I conduct this online exam, I was wondering what are they doing at the other side. Whether they are actually discussing among themselves or get the help from other people, we wouldn't know.” (P17)* *“Of course at the same time we have to inculcate academic honesty…. I think is to start by letting them know their rights and what academic honesty is all about, what intellectual property is all about, you know. Then to have a certain sense of pride in their own learning and their own attainment.” (P20)*
Cost	IT softwares	*“Ideally we should have a really good system so that we can do the questions online and the proctoring.” (P2)*
	Internet connection	*“I think some of them face challenges with the internet connection, I think that is the main issue because of stability of internet. And some of the students … when they are downloading their questions, take a bit of time to download the questions. (P10)*
	Technical support	*“I think also having the IT people and the eLearning people present when all these things (issues) happen … I think they were really important.” (P2)* *“If they closely monitor, all these (technical issues will be actually resolved, can be resolved immediately.” (P6)*
	Faculty time	*“I think on the faculty opinion right, normally they will prefer to have the on campus exam…It's just mainly on the time that the faculty has to spend to set higher order thinking questions.” (P10)*

#### Validity

The interviewees claimed that the validity of ROOBE depended on assessment blueprinting, faculty competencies in item writing as well as quality assurance of examinations. Most of them agreed that it was suitable for assessing learning outcomes that focused on higher order thinking, especially for clinically oriented topics such as pathology and microbiology. They highlighted its limitation for use in pre-clinical semesters whereby the learning outcomes were largely based on recalling and understanding of knowledge. Preference could be given to low stakes and in-course assessments. Besides multiple-choice questions, short answer questions and essays, it could also be considered for take home examinations, where students are given a set period of time to complete an assessment task. A shorter examination duration has been proposed to prevent the students from having ample time to search for the answers from the available resources during ROOBE. Nevertheless, ROOBE was less appropriate for assessment of clinical and practical skills.

Faculty competencies in setting higher order questions were important to ensure the validity and quality of the examinations. These were achieved through faculty workshops as well as on-the-job training. The faculty obtained useful feedback through the quality assurance activities including vetting of examination papers and post-examination item analysis.

#### Reliability

The interviewees’ views were varied regarding the reliability of ROOBE. Some felt that the student performance was comparable to that in conventional CBE, while some commented that students performed better in ROOBE which could be attributed to students’ better preparedness due to the perception that the questions could be more challenging. Nevertheless, it was highlighted that students who had good typing skill could be advantaged compared to the others during ROOBE. Standard setting was identified as a crucial exercise to establish the appropriate standard in ensuring the student competencies.

#### Education impact

The interviewees noted that ROOBE promoted students’ higher order cognitive skills including critical thinking, decision making and problem solving as the questions were designed to test these competencies. They opined there would be a shift in how students prepared for the examinations, from memorisation of facts to application of knowledge. Meanwhile, they acquired skills to access, organise and interpret information. These could contribute to the students’ work readiness.

#### Acceptability

The transition from CBE to ROOBE involved change management from the perspectives of faculty and student readiness. There was initial resistance from some faculty due to fear of uncertainty, but this reduced as they gained experience in implementation and with the support of faculty training in setting questions as well as availability of literature evidence. A number of them highlighted that the COVID-19 pandemic has necessitated ROOBE and shortened the transition period.

The interviewees highlighted that it was crucial to have clear guidelines on the conduct of ROOBE and contingency plans in place should technology fail during a ROOBE. In addition, technical support on the use of the online assessment system as well as during conduct of ROOBE was important. This would help to boost their confidence in conducting ROOBE.

Some interviewees expressed their concerns about uncertainty with students’ academic integrity during ROOBE and hence suggested proctoring. Meanwhile, they opined that academic honesty should be inculcated among the students. Other concerns were around the acceptance and recognition of ROOBE by regulatory bodies, employers and the public as it was a relatively new tool.

#### Cost

The interviewees shared their views that the user experience with ROOBE depended on the efficiency of the online assessment platform and internet connection. Besides, online proctoring softwares were suggested. Technical support should be provided to ensure that technical issues could be resolved timely. In addition, they highlighted that faculty spent a large amount of time to prepare the higher order questions for ROOBE which some considered as additional workload.

### Students’ perceptions of ROOBE

A total of 249 students (170 males, 79 females) participated in the questionnaire (32% overall response rate). Of these 249 respondents, 26.5%, 14.9%, 41.8% and 16.9% were from Semesters 2 (year 1), 3 (year 2), 4 (year 2) and 5 (year 3), respectively.

**Fig. 1:**
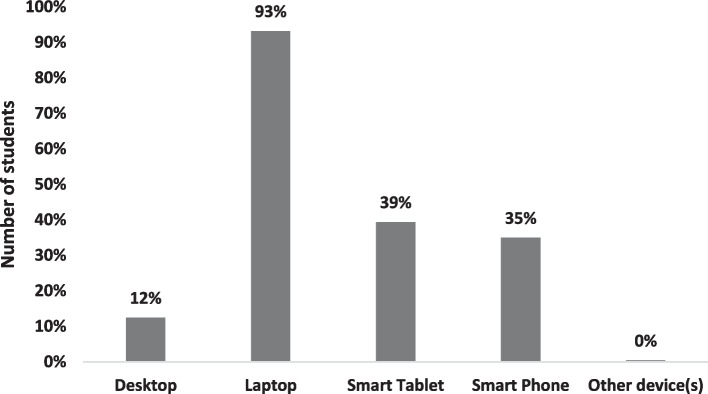
Types and frequencies of devices used by students during ROOBE

Laptop computers were most commonly used by the students during ROOBE, followed by smart tablets and smart phones (Fig. 1). While 40% of them used a single device, 40% and 20% of them used 2 and 3 devices, respectively, during the examinations.

**Fig. 2:**
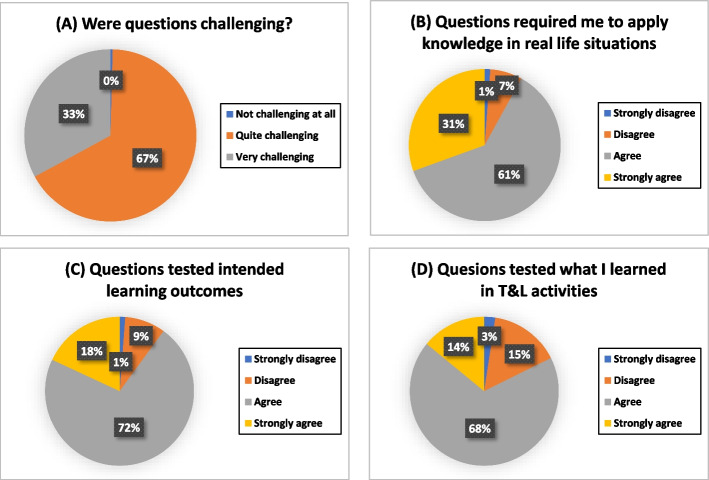
Students’ perceptions of the questions in ROOBE

All the participants found the questions in ROOBE challenging, and 92% of them perceived that the questions assessed their ability to apply knowledge in real life situations. Majority of them agreed that the questions assessed the intended learning outcomes and what they learned in the teaching and learning activities. The details are presented in Fig. 2. About two third of the students claimed that the formative assessments and mock examinations were useful in helping them to prepare for the actual examinations.

**Fig. 3:**
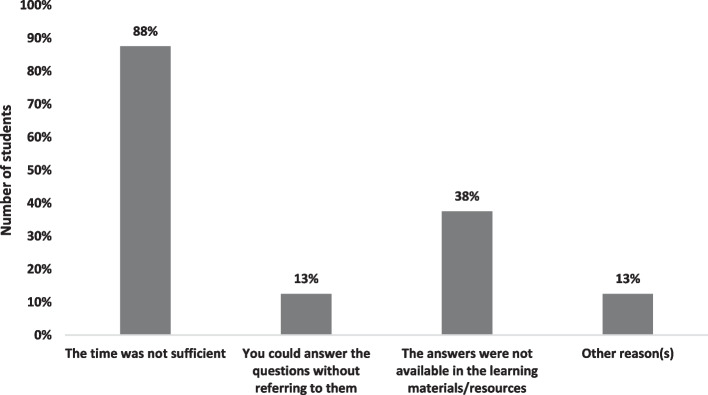
Students’ reasons for not referring to any learning resources during ROOBE

Only 3% of the students did not refer to any learning resources during ROOBE. The reasons are shown in Fig. 3. For those who utilised the references, they served the purpose of helping the students to refresh their memory (84%), find answers to the questions (71%) and find calculation method or formula (12%). The rest of them (5%) used these to confirm their answers, find the meaning of difficult words or clarify difficult concepts.

**Fig. 4:**
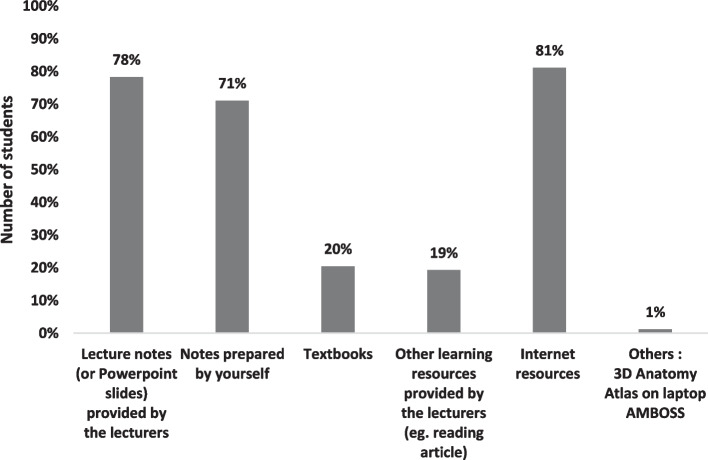
Types of learning resources referred during ROOBE

**Fig. 5:**
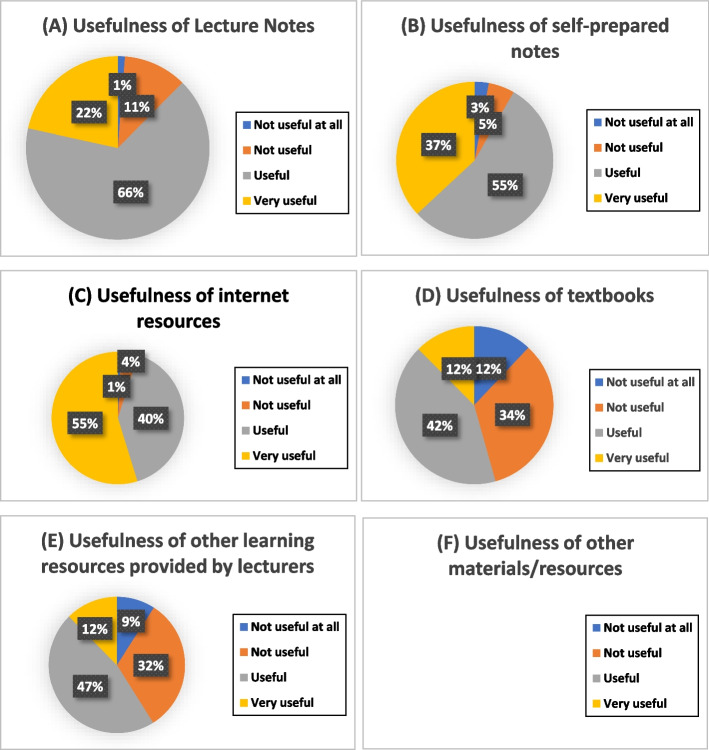
Students’ responses on the usefulness of various types of learning resources

The students referred to various types of learning resources during ROOBE, as shown in Fig. 4. Internet resources, lecture notes and self-prepared notes were the most frequently used references. They were also rated by more than 85% of the participants as the most useful resources during ROOBE (Fig. 5). The survey showed that 78% of the participants managed to find some of the answers and 13% could find most of the answers from the resources accessed.

**Fig. 6:**
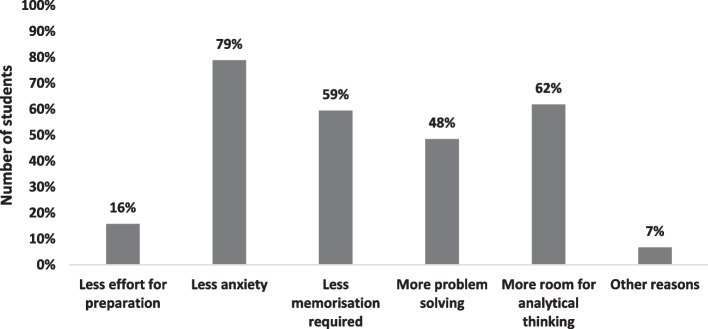
Reasons for students’ preference for ROOBE

Two third of the students expressed that they preferred ROOBE to CBE held in examination hall under in-person invigilation. Specifically, the percentages of students in years 1, 2 and 3 who preferred ROOBE to CBE were 67%, 69% and 60%, respectively. The reasons are shown in Fig. 6, mainly due to less anxiety, more room for analytical thinking and problem solving as well as less memorisation required. Other reasons include less disturbance and concerns for COVID-19 infection if the examinations were held in the examination hall.

The students who did not prefer ROOBE elaborated that the questions in ROOBE were more challenging as they tested application of knowledge. Consequently, they did not have sufficient time to search for the information in the resources. Some were also concerned that the lack of memorisation of knowledge and dependence on information resources might reduce their confidence for future practice. A few students also cited that they were less motivated to study for ROOBE as they assumed that they could search for the answers from the learning resources during the examinations. The other comments related to the negative experience which included disruption in internet connection and noise disturbances when the other examinees asked questions during an invigilated ROOBE. Several students have highlighted the concern of possible cheating among the examinees during ROOBE whereby the students might communicate with one another using various means. Nevertheless, this could be individual perception, as one student wrote:

I was concerned about cheating incidents but after taking a few exams I think the questions were designed in a way to make us beat the clock hence I don’t think it’s very possible for the cheaters to waste time.

Despite the concerns, ROOBE were perceived positively by the students. They suggested a more efficient invigilation process to improve the examination experience. This included reducing the number of software applications that they were required to log in during ROOBE, which could be a possible cause for lagging issues with internet connection. One student commented that conducting the examinations in the campus could ensure equitable internet speed for all candidates and better technical support for the students during ROOBE.

There were also suggestions to help students to be better prepared for OBE, including aligning the syllabus and learning materials to OBE as well as providing more practice questions that assess higher order cognitive skills. More clinical scenario-based questions were considered useful. Time allocation for each question should be carefully considered. While the examination duration should be limited to prevent students from having time to research or discuss their answers with their peers, it should be sufficient for them to check the allowed resources.

## Discussion

Validity, educational impact and acceptability were the predominant assessment considerations by the faculty interviewees with regards to ROOBE. Those who had experience with the conduct of ROOBE highlighted that assessment blueprinting was crucial in guiding the design of questions in ROOBE according to the intended learning outcomes. They agreed that ROOBE was especially useful for assessing higher order cognitive skills and faculty competencies in writing these questions could be achieved through faculty development activities such as faculty guides, workshops and on-the-job training. Quality assurance activities including peer review and vetting of questions also played important roles in ensuring the validity of the examinations.

The other aspect that was highlighted during the interviews was the acceptability of ROOBE by the stakeholders including the regulatory bodies, employers and the public. The issues discussed were mainly related to the concerns on students’ academic integrity during the examinations, particularly about the potential risk of students sharing answers with each other which could be difficult to monitor online. Similar concerns have been shared by academics globally as evident from the literatures [13, 20, 21]. These issues could affect the stakeholders’ confidence on the quality of the graduates. Inevitably, change management is necessary to ensure the faculty and students’ readiness for open book examinations which entails its principles and objectives, the technology platform as well as a comprehensive guide detailing the contingency procedures should unexpected incidences occur during the examinations. The change recipients i.e. faculty, students, professional staff in examination management and external stakeholders should be engaged in the discussion from the early stage and have opportunities to express their views and concerns related to ROOBE implementation. It is important to have effective leaders in different roles who are united by the common stance and way of thinking to handle the change confidently at the organisation and personal level [22].

The faculty’s views about the reliability of ROOBE were varied. This could be influenced by the quality of the items in the examination, students’ preparedness for the types of questions in open book examinations via formative assessment and practice, as well as other factors that might affect the conduct of the examinations such as the students’ typing skills and technical glitches using the online examination platform. Investment in IT infrastructure, softwares and technical support would be necessary to enhance the user experience. These were the associated cost for the conduct of ROOBE on an online platform. Meanwhile, the interviewees commented that faculty spent considerably larger amount of time to prepare the higher order questions for an open book examination. The findings suggested that reliability and cost could be the trade-offs for ROOBE. We argue that compromise on reliability in favour of the educational impact of the assessment is acceptable, which is the essence of the conceptual framework of assessment utility [18].

The medical students in this study perceived that OBE were more challenging as they were assessed on their higher order thinking skills. More than half of the survey participants preferred this type of examination over CBE due to less anxiety, more room for analytical thinking and problem solving as well as less memorisation required. This was consistent with the findings in another study among pharmacy students [6]. Lecture notes, self-prepared notes and internet resources were cited as the most frequently referred and useful resources during the examinations, mainly to refresh their memory and find answers to the questions. Despite this, many found that the examination duration was insufficient for them to search for the answers. Some students might assume that they could find the answers in the resources during the OBE and hence inadequately prepared for the examination. To address this issue, it is essential to provide adequate practice questions and student guide for the students in light that assessment drives learning. The students’ feedback that formative assessment and mock examination helped them in preparation for OBE. While OBE encourages testing of application of knowledge which is appreciated by the students, the objective is not to downplay the role of factual memorisation as a foundation for higher order thinking and problem solving. Memorising and understanding are not mutually exclusive in the learning process [23]. In a study by Pandey and Zimitat [24], a combination of memorisation (surface learning approach) with understanding and visualisation (deep learning approaches) led to successful learning of anatomy among medical students. The current study has shown that 88% of the students found the self-prepared notes useful during the OBE. Note taking is a form of engagement with the learning materials that has been shown to promote deeper understanding [25].

The other concerns highlighted by students were attributed to the technical challenges associated with the conduct of the ROOBE on an online platform remotely. These include internet connection, computer hardware and software glitches which could affect the user experience during the examinations. According to the cognitive load theory [26], these could cause undesirable cognitive load on the students during the examinations that might compete with their working memory. Therefore, it is important to enable the students to rehearse the ROOBE procedures prior to the examinations and provide immediate technical support during the examinations [27]. With regards to academic integrity issues brought up by the students during ROOBE such as collusion, these should be dealt with according to the procedures for handling student misconduct cases. Invigilation for a remote online examination is resource intensive without the use of online proctoring services. On the other hand, there are risks associated with artificial intelligence (AI) based proctoring systems, especially ethical concerns with regards to student privacy and personal data use, as well as potential academic unfairness associated with AI-informed judgement [28]. These could also create mistrust among the students toward the academic institution. Instead, the academic institution should invest in strategies to instill academic integrity among the students which will be more beneficial in the long term.

### Limitations of the study

The study was carried out during the COVID-19 pandemic period. Due to the restrictions in access to hospitals during the period, posting and examinations in the clinical years of the medical programme were postponed. ROOBE examinations were conducted for the basic sciences only. Therefore, the student survey data were collected among the pre-clinical students. Besides, it was a learning phase for some faculty in writing higher order questions as well as the use of the online examination platform during the pandemic. Although they were provided with training and support, the lack of experience could affect their perceptions about ROOBE. Nevertheless, the findings from this study are useful lessons for others who intend to implement ROOBE given that diverse faculty demographics is common in most institutions.

## Conclusions

Education institutions are facing constant pressure in adaptation and innovation; however, stakeholders’ resistance is commonly faced. The COVID-19 pandemic has been a key catalyst for the acceptance of ROOBE, whereby the initial fear of uncertainty has gradually been replaced with increase in confidence as the faculty and students gain more experience and readiness through repeated implementation of ROOBE during the pandemic lockdown period. It was encouraging to observe that both the educators and students recognised the potential of OBE to navigate the students towards deep learning approach and higher order cognitive skills, which in turn would better prepare them for the fast-changing healthcare work environments. The concerns related to the technical challenges and academic integrity issues in ROOBE are not to be neglected, however, they can be addressed with appropriate change management, training and support. In line with the 2018 Consensus Framework for Good Assessment [29], the findings of this study suggest that ROOBE is an appropriate tool in the systems of assessment approach for producing competent healthcare professionals. It continues to be relevant post-pandemic in view of the rapidly changing education landscape and advancement of technologies.

As generative AI technologies gain pace and become widely accessible to everyone, it is essential to direct further research efforts to explore their benefits, risks and ethical considerations in ensuring the validity and reliability of ROOBE. While universities are responsible to equip graduates with skills in emerging technologies, students need to be educated on how these tools work as well as their unintended consequences. Meanwhile, generative AI may potentially assist faculty in the design of ROOBE for assessment of critical thinking skills. In short, comprehensive evidence from various perspectives will help educators to enhance the utility of ROOBE in delivering a fit-for-purpose assessment.

## Data Availability

The data supporting the conclusions of this article are included within the article.
